# Book Review: The Emotional Rollercoaster of Language Teaching

**DOI:** 10.3389/fpsyg.2020.593312

**Published:** 2020-10-19

**Authors:** Wenxiu Chu, Honggang Liu

**Affiliations:** School of Foreign Language, Northeast Normal University, Changchun, China

**Keywords:** language teacher, emotion labor, emotion regulation, well-being, resilience, anxiety

While emotion, as a key factor in positive psychology (Moneta, 2014), has long been researched in the psychological field, it has received less attention in the field of language teacher education (King and Sarah Ng, 2018; Martínez Agudo, 2018). The “emotional turn” (White, 2018) in the field of language teacher education, however, has led to a call for more attention to the language teacher emotion (LTE). The editors of the volume under review respond to this call in an exploration of the complexity and dynamism of LTE.

This edited book consists of 16 chapters. The first chapter introduces LTE and presents the collection's purpose and organization. The final chapter, Chapter 16, reviews the collection's research findings and methods, explains the definition of “emotional rollercoaster,” and emphasizes the role of emotions and emotion regulation in language teacher training and professional development. The rest of the volume consists of empirical studies that can be divided into three thematic groups. The first group (Chapters 3, 4, 8, 9, and 12) includes representative topics about teacher emotions. The chapters discuss the emotional labor of foreign language teachers in the US public school system (Chapter 3) and at a US university (Chapter 4), the emotional experience of English teachers in six tertiary education programs in the United States and United Kingdom (Chapter 8), and, finally, the emotional labor and burnout of Japanese language teachers (Chapter 9) and Nepali English teachers (Chapter 12).

The second thematic strand (Chapters 2, 5, 6, 7, 10, 14, and 15) concerns cognitive psychological factors related to teacher emotions. The chapters consider the subjective well-being of CLIL teachers in Austria (Chapter 2); language teacher identity and emotion in an action research program (Chapter 5); the resilience of pre-service language teachers in their first practicum (Chapter 6); past L2 selves, emotions, and classroom group dynamics (Chapter 7); the anxiety of Japanese elementary school English teachers in a training intervention program (Chapter 10); the emotional well-being of language teachers (Chapter 14); and EFL/ESL teachers' motivation and emotional intelligence (Chapter 15). The third thematic group (Chapters 11 and 13) comprises strategy and intervention studies on teacher emotional regulation, which primarily examine the emotion regulation behavior of experienced EFL teachers in Japan (Chapter 11) as well as the stressors of language teachers and functions of intervention (Chapter 13). This unique book about LTE is highly worth reading. Its contributions lie in the following three aspects. First, it performs pioneering work by interweaving classic topics, such as emotional labor and intelligence, and cutting-edge themes, such as L2 selves and teacher well-being, in the field of language teacher education. It draws transdisciplinary support from psychology and teacher psychology, in which teacher emotion has long been researched. Emotional labor, emotional intelligence, and an array of psychological factors (e.g., burnout, identity, and self-efficacy) pertinent to LTE have become central research topics in teacher psychology and, consequently, are regarded as the core themes in language teacher education. This collection explores many psychological factors pertaining to emotions, including well-being, resilience, and motivation and highlights the impact of previous emotion research on LTE. It also points to future research directions for scholars interested in those topics.

The collection investigates novel research topics in language teacher psychology as well, including the L2 self. For example, Chapter 7 dwells on the relationships between past L2 selves, emotions, and classroom group dynamics. The chapters reflect contemporary research trends in LTE, such as relationships between teacher emotional labor, emotional intelligence, emotional regulation, well-being, and identity. Another strong point of the volume is its exploration of the dynamism and complexity of LTE in diverse cultural contexts; the chapters concentrate on LTE in various countries and areas, including Australia, America, Japan, Nepal, and West Africa, and involve elementary school, secondary school, and university language teachers as well as pre-service language teachers. The exploration of why and how teacher emotions fluctuate over time is of great benefit to recognizing common stressful events in language teaching and preparing teachers to resist mental stress and regulate their emotions. For example, Chapter 2 examines subjective well-being and its influencing factors of Australia CLIL teachers using qualitative research methods, and Chapter 12 analyzes the influencing factors of language teacher's emotional labor in Nepal.

Multiple research methods are used to investigate LTE in this volume, and this diversification of research methods should be given significant attention. Qualitative methods are often used for LTE research due to its changeability and intricacy (Xu, 2018). This methodological tendency is also reflected in this book, in which 14 chapters are empirical studies and 10 adopt qualitative research methods. However, quantitative research methods, such as questionnaires or scales, are also utilized to examine some emotion-related topics in general education, such as teacher emotional labor (e.g., Yin et al., 2019). In this volume, two studies adopt quantitative research methods. Furthermore, mixed-methods research, combining the strengths of quantitative and qualitative methods, can probe the complicated processes of teacher emotions, making it a particularly promising research method. It is utilized in two chapters of this book.

Finally, the research on emotion regulation in this collection provides strategic guidance for language and non-language teachers to navigate negative emotions and challenges. Language teachers experience many pressures originating from institutions, colleagues, and students. Indeed, language teaching is a profession full of crises (Hiver and Dörnyei, 2015), making it crucial to explore emotion regulation in the field. The empirical discussion about emotion regulation strategies guides teachers in regulating their emotions and maintaining a positive attitude and professional commitment. For instance, Chapter 11 analyzes four types of strategy—related to situation, attention deployment, cognitive change, and response modulation—for handling teaching adversities.

Providing fundamental insights into the complexity of teacher emotions, this collection is devoted to exploring the role of these emotions in diverse cultural contexts by combining emotion theory, emotion-related psychological factors, and language teaching practice. It is suitable for anyone interested in learning about and further investigating teacher emotions.

## Author Contributions

WC and HL chose this book together. WC wrote the review. HL provided valuable guidance for the draft. All authors contributed to the article and approved the submitted version.

## Conflict of Interest

The authors declare that the research was conducted in the absence of any commercial or financial relationships that could be construed as a potential conflict of interest.
